# RETRACTION: Assessing the Efficacy of Naoxintong Capsules on Wound Healing in Post‐Craniotomy Patients: A Clinical Perspective

**DOI:** 10.1111/iwj.70538

**Published:** 2025-04-18

## Abstract

**RETRACTION:** ZhengG. , YuT. , HumayunA. , and ChenH. , “Assessing the Efficacy of Naoxintong Capsules on Wound Healing in Post‐Craniotomy Patients: A Clinical Perspective,” International Wound Journal 21, no. 3 (2024): e14806, 10.1111/iwj.14806.38414325 PMC10899796

The above article, published online on 27 February 2024, in Wiley Online Library (http://onlinelibrary.wiley.com/), has been retracted by agreement between the journal Editor in Chief, Professor Keith Harding; and John Wiley & Sons Ltd. Following an investigation by the publisher, all parties have concluded that this article was accepted solely on the basis of a compromised peer review process. In addition, the investigation found that the authors provided incomplete ethical approval information for the study. The editors have therefore decided to retract the article. The authors did not indicate their agreement with the retraction.

**RETRACTION:** G. Zheng , T. Yu , A. Humayun , and H. Chen , “Assessing the Efficacy of Naoxintong Capsules on Wound Healing in Post‐Craniotomy Patients: A Clinical Perspective,” International Wound Journal 21, no. 3 (2024): e14806, 10.1111/iwj.14806.38414325 PMC10899796

The above article, published online on 27 February 2024, in Wiley Online Library (http://onlinelibrary.wiley.com/), has been retracted by agreement between the journal Editor in Chief, Professor Keith Harding; and John Wiley & Sons Ltd. Following an investigation by the publisher, all parties have concluded that this article was accepted solely on the basis of a compromised peer review process. In addition, the investigation found that the authors provided incomplete ethical approval information for the study. The editors have therefore decided to retract the article. The authors did not indicate their agreement with the retraction.

